# Experimental validation of novel genes predicted in the un-annotated regions of the Arabidopsis genome

**DOI:** 10.1186/1471-2164-8-18

**Published:** 2007-01-17

**Authors:** William A Moskal, Hank C Wu, Beverly A Underwood, Wei Wang, Christopher D Town, Yongli Xiao

**Affiliations:** 1 The Institute for Genomic Research, 9712 Medical Center Drive, Rockville, Maryland, 20850, USA

## Abstract

**Background:**

Several lines of evidence support the existence of novel genes and other transcribed units which have not yet been annotated in the Arabidopsis genome. Two gene prediction programs which make use of comparative genomic analysis, Twinscan and EuGene, have recently been deployed on the Arabidopsis genome. The ability of these programs to make use of sequence data from other species has allowed both Twinscan and EuGene to predict over 1000 genes that are intergenic with respect to the most recent annotation release. A high throughput RACE pipeline was utilized in an attempt to verify the structure and expression of these novel genes.

**Results:**

1,071 un-annotated loci were targeted by RACE, and full length sequence coverage was obtained for 35% of the targeted genes. We have verified the structure and expression of 378 genes that were not present within the most recent release of the Arabidopsis genome annotation. These 378 genes represent a structurally diverse set of transcripts and encode a functionally diverse set of proteins.

**Conclusion:**

We have investigated the accuracy of the Twinscan and EuGene gene prediction programs and found them to be reliable predictors of gene structure in Arabidopsis. Several hundred previously un-annotated genes were validated by this work. Based upon this information derived from these efforts it is likely that the Arabidopsis genome annotation continues to overlook several hundred protein coding genes.

## Background

A complete annotated genome sequence of Arabidopsis thaliana was released by the Arabidopsis Genome Initiative (AGI) in the year 2000, the first completed plant genome[1]. Since then, our understanding of the Arabidopsis genome structure and transcriptome has been improved through the release of 4 sequential updates to the annotation, culminating in The Institute for Genomic Research's release 5 (TIGR5), which forms the basis of the work presented here. Following the TIGR5 annotation release, responsibility for maintaining and updating the Arabidopsis annotation was turned over to The Arabidopsis Information Resource (TAIR), which has since released version 6 of the Arabidopsis annotation (TAIR6). Over the course of the TIGR annotation releases, the number of annotated protein-coding genes of Arabidopsis has increased from 25,498 (a number that included transposons and pseudogenes) to a final total of 26,207 protein coding genes plus 3,786 regions annotated as transposon-related or other pseudogenes in the final TIGR release. At the same time, the size of the Arabidopsis pseudomolecules has increased from 115 MB in the initial 2000 release, to 119 MB in TIGR5 due to the inclusion of additional finished and unfinished BACs.

While the sequential TIGR re-annotations of the genome have been relatively stable in terms of overall gene density and gene structure statistics, the major benefits of the re-annotation efforts have come from the incorporation of expressed sequence tags (ESTs) and full length complementary DNA (FL-cDNA) clone sequences into the Arabidopsis annotation, improving the accuracy of individual gene structures [2-4]. However, transcripts from the most lowly expressed genes, or genes specifically expressed in important but relatively minor cell types such as meristems or the Arabidopsis gametophyte stage may very likely be under-represented in the over half million ESTs available through GenBank. To provide experimental support for genes lacking EST or other cDNA evidence, we have previously carried out high-throughput Rapid Amplification of cDNA Ends (RACE) experiments and generated partial or complete sequence for over 1000 genes, leading to the improvement of many gene structures [5,6].

Genome annotation is never complete or final. Since its release in January of 2004, various lines of evidence have come to light which suggest that the TIGR5 annotation still paints an incomplete picture of the Arabidopsis gene space and transcriptome. Continued submission of ESTs and other sequence information to GenBank reveals the existence of transcripts that do not map to currently annotated genes [7,8]. These may represent novel protein coding genes, genes which code small unknown peptides, or may also represent non-coding RNA. Additionally, evidence of transcription in un-annotated intergenic regions of the genome has been seen through Massively Parallel Signature Sequencing (MPSS) efforts which reported several thousand transcript signatures from un-annotated intergenic regions [9]. Analysis of whole-genome tiling arrays to examine the Arabidopsis transcriptome have also provided strong indications for the presence of over five thousand novel transcriptional units [10,11]. A survey of the Arabidopsis genome for a family of divergent cysteine rich anti-microbial defensin-like peptides yielded over 300 genes, 80% of which were absent from TIGR's Arabidopsis annotation [12].

The wealth of new sequence data for other plant species that has become available since the landmark release of the Arabidopsis genome has now allowed for the refining and improvement of gene detection based upon comparative genomic analysis. Comparative genomics techniques have been proven extremely valuable for identifying conserved genes and regulatory elements in a variety of closely related species and has been already been applied effectively to the human genome [13-15], as well as the malaria parasite genome [16] and the *C. elegans *genome [17], among others. The Arabidopsis genome annotation is also beginning to benefit from comparative genomic analysis. A comparative study of *Arabidopsis thaliana *and *Brassica oleracea *yielded a large number of Conserved Arabidopsis Genome regions (CAGS), 72% of which aligned with predicted genes [18]. The remaining intergenic CAGS suggest the existence of several thousand currently un-annotated genes. RACE experiments have demonstrated transcriptional activity at 58 of 192 targeted CAGS, demonstrating that the CAGS may correspond to conserved un-annotated genes. A separate, similar investigation comparing the same *Brassica olreacea *sequence set with the Arabidopsis genome resulted in the identification of 25 genes that were missed by the TIGR annotation [19].

Two relatively new gene prediction tools that make use of comparative genomics have been deployed for analysis of the Arabidopsis Genome: Twinscan [20] and EuGene [21]. Twinscan is a gene-structure prediction program that extends the probabilistic models employed by the *ab initio *gene finding program GENSCAN [22]. Twinscan exploits cross-species homology between closely related genomes to produce improved gene models. EuGene is another gene prediction program developed to make use of comparative genomics for improved gene models. EuGene makes use of multiple homologous sequences (including ESTs, protein sequences and genomic homologous sequences) from closely related organisms, tblastx analysis, splice site analysis and probabilistic models to provide gene predictions. Both the Twinscan and EuGene programs have been applied to the Arabidopsis genome, resulting in predictions for 30,635 and 28,530 protein coding genes, respectively. Both Twinscan and EuGene predict more genes than currently exist in the TIGR5 annotation, and there is significant overlap between the predictions produced by each program and with the TIGR5 annotation. It is only within the more challenging un-annotated regions of genome where less evidence exists to support gene predictions where the overlap between Twinscan and EuGene predictions begins to decrease significantly. In addition to this work, Twinscan has been applied broadly and with success to refine the annotation of the *C. elegans *[23], chicken [24], and rat [25] genomes. The target for EuGene at present is plant genomes and in addition to Arabidopsis, has been deployed for poplar [26] and barrel medic [27].

Like the hypothetical genes of Arabidopsis that we have studied previously [5,6], most of the novel genes predicted by Twinscan and EuGene lack experimental support. To assess the validity of these Twinscan and EuGene predictions we have applied a high-throughput RACE pipeline and have verified the presence, structure, and expression of several hundred currently un-annotated genes, of both deducible and potentially novel function.

## Results and discussion

### Intergenic predictions

Several lines of evidence indicated that many likely genes were not captured by the TIGR5 annotation. However, generating experimental evidence for these genes and their structures by RACE requires a working model upon which to design primers. Pilot experiments based on Twinscan and EuGene predictions, as well as other evidence (Hu and Brent, personal communication) showed that primers designed upon either Twinscan or EuGene predictions had good success rates, whereas primers designed against CAGS performed relatively poorly. Therefore, we focused our efforts on genes predicted by one or both of these programs. In TIGR5 intergenic space, defined here as all regions of the genome which do not overlap on the same strand with any annotated genes, there were 1,515 Twinscan predicted genes and 1,774 Eugene predicted genes, with the intersection of these 2 sets being 365 loci. The gene sizes and exon statistics for these genes are summarized in Table 1. Interestingly, EuGene predicts a larger number of smaller "genes" including 239 spliced predictions with predicted CDS lengths of less than 100 bp. Surprisingly, the average size of an intergenic spliced EuGene prediction is smaller than that of an intergenic single exon (EuGene) prediction. A significantly higher percentage of intergenic Twinscan predictions have CDS sizes of over 300 bp than do the intergenic EuGene predictions. We targeted 1,071 intergenic regions with our RACE pipeline. Four hundred and forty eight (448) primer pairs were designed that were expected to amplify a gene predicted only by Twinscan. Three hundred and forty five (345) primer pairs were designed that were expected to amplify a gene predicted only by EuGene. An additional 278 primer pairs were designed which were compatible with overlapping predictions generated by both Twinscan and EuGene in the same genomic region.

### RACE success rates

**Figure 1:**
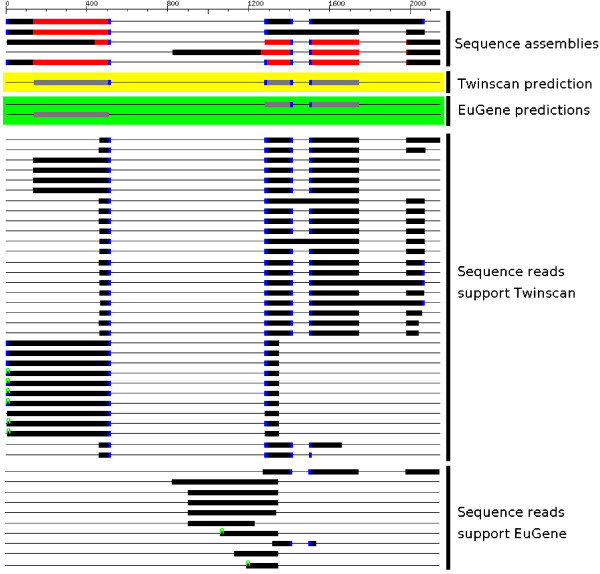
**Merging of EuGene predictions**. At this locus, Twinscan predicts a single large 3 exon ORF (yellow), while EuGene splits this gene to predict 2 smaller ORFs within the same frame (green). A minimal set of sequence assemblies generated by PASA are shown with ORFs shown as red. We have recovered experimental evidence supporting transcription of both the merged ORF as best predicted by Twinscan and one of the smaller ORFs, as better predicted by EuGene. Poly A tail locations are denoted by a green 'A'.

The success of the RACE pipeline, defined as the frequency of obtaining RACE product(s) that mapped to an intergenic prediction (regardless of how well the experimental evidence agreed with overlapping predictions), is summarized in Table 2. Both Twinscan and EuGene predicted genes with good efficiency. We obtained full length experimental sequence support, either from RACE data or subsequent full length cloning attempts, for 378 genes out of 1,071 targeted. Two hundred and fifty seven (257) of the verified genes overlapped with at least one EuGene prediction, 304 overlapped with at least one Twinscan prediction, and 164 genes overlapped with at least one or more Twinscan and one or more EuGene predictions. In several instances, our experimentally verified transcript assemblies overlapped multiple Twinscan or EuGene predictions, such as neighboring genes At.chr4.2.13 and At.chr4.2.14. We also observed instances where our experimental results merged the two neighboring gene predictions into a single ORF, as was the case with EuGene predictions At02eug07640 and At02eug07630 (Figure 1). Interestingly, in the case of these 2 EuGene predictions, while most of our experimental data suggests a longer ORF that was better predicted by Twinscan than EuGene, we have also identified several clones which posses polyA tails and support one of the shorter, unmerged ORFs predicted by EuGene. This suggests that Twinscan and EuGene may have independently predicted different isoforms of the same gene.

With our RACE pipeline, we observed full-length success rates of 42% for genes predicted by Twinscan, 41% for genes predicted by EuGene, and a much higher 58% for genes predicted by both programs. We also obtained partial length sequence from an additional 49 genes. These genes were determined to be partial length due to the absence of either a START codon, a STOP codon, or an intact open reading frame relative to the underlying prediction.

### Structure of novel genes

**Figure 2:**
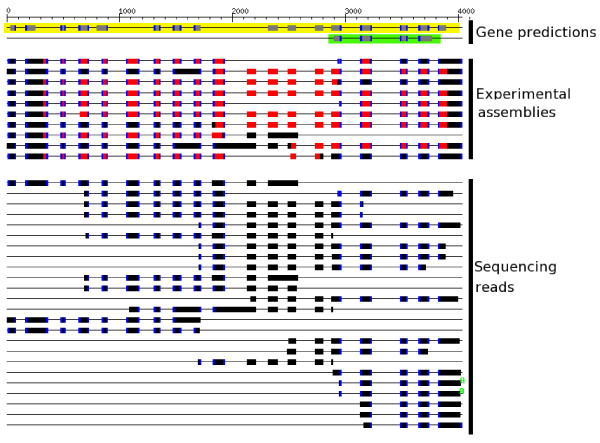
**Example of a previously unpredicted gene**. This region contains a EuGene prediction, At01eug01210 (green), a Twinscan prediction At.chr1.1.117 (yellow). Sequencing reads are shown in black. A minimal set of sequence assemblies are also shown with potential ORFs highlighted in red. Conserved splice junctions are shown as blue bars. PolyA tail locations are denoted by a green 'A'.

**Figure 3:**
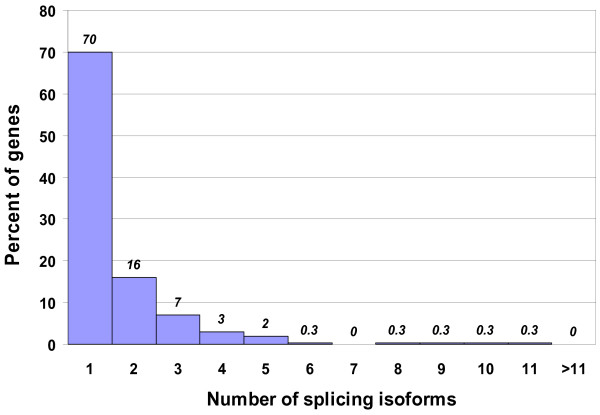
**Extent of alternative splicing of previously unannotated genes**. Number of isoforms per 100 genes.

The novel genes validated by our RACE pipeline vary widely coding length and exon count (Table 2). Although many of the novel genes were relatively short and contained a single exon and un-spliced transcript, there are some striking exceptions. Figure 2 shows Twinscan predicted At.chr1.1.117, a gene for which we recovered 10 splice isoforms, possessing between 7 and 18 coding exons. Alternative splicing is observed with over 30% (113/378) of the un-annotated genes verified through these efforts. Genes were detected having between 1 (265) and 11 (1) splicing isoforms (Figure 3).

### Accuracy of Twinscan and EuGene predictions

**Figure 4:**
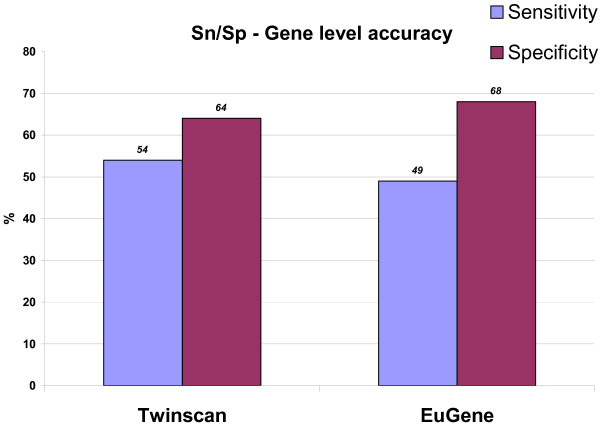
**Structural accuracy of Twinscan and EuGene predictions**. Gene level Sensitivity (Sn) and Specificity (Sp) were calculated using GTF files generated from BLAT alignment coordinates and the Eval software package.

The gene level performance (sensitivity (Sn) and specificity (Sp)) of the Twinscan and EuGene predictions was determined using as a reference set the longest experimentally verified open reading frame from each of the 378 genes for which we recovered full length sequence, comparing these with only those intergenic predictions which overlapped this set. This analysis included 21% of the total intergenic Twinscan predictions and 15% of the total intergenic EuGene predictions. These data are summarized in Figure 4. Sensitivity (probability that a feature that is known to exist is correctly predicted) and specificity (probability that a predicted feature is correct) is shown at the gene level. On this level, both EuGene and Twinscan performed similarly, with sensitivities (percent of validated genes that are correctly predicted) of 49% and 54%, respectively. The specificities were also similar for both programs.

### Functional analysis of un-annotated genes

To investigate the possible functions of these un-annotated genes, we searched 378 intergenic full length ORF sequences against TIGR's in-house non-identical protein database using blastx. Two hundred and seventy eight genes had significant protein matches. Approximately 50% (141/278) of the genes having a significant database hit are most similar to hypothetical proteins, or other proteins of unknown function. Many genes were also found to have significant matches with well characterized proteins. The top ten blast hits are shown in Table 3. Several genes such as Twinscan predicted At.chr1.1.117 (Figure 2), which aligns with a sub-family of alpha 1,6, mannosyltransferase enzymes, are not similar to any annotated Arabidopsis genes. This gene is conserved across the Eukarya with homologues from human, mouse, zebrafish, yeast, and rice. Others, such as At.chr1.1.48 are highly similar to hypothetical genes in Arabidopsis. Multiple sequence alignments with homologues of this gene show that it is a member of a sub-family of uncharacterized hairpin domain containing proteins that is specific to Arabidopsis, suggesting more recent duplication events.

### Expression patterns of un-annotated genes

**Figure 5:**
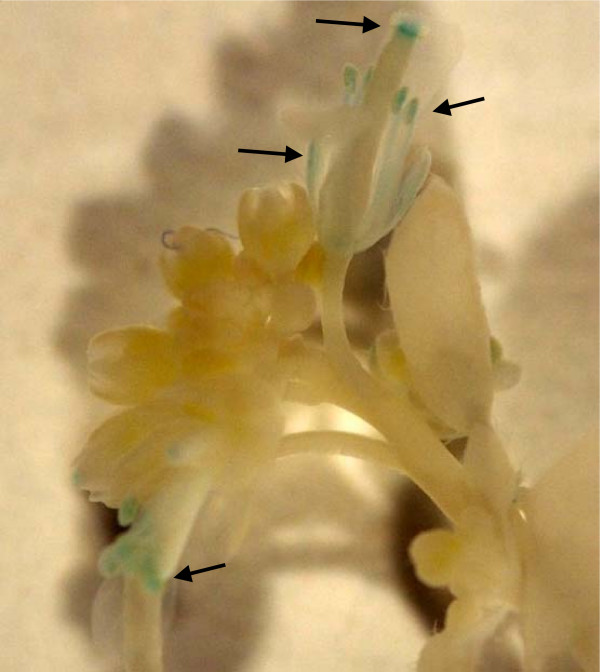
**Expression pattern of At.chr1.16.98**. GUS staining pattern observed for enhancer trap line ET7211, which tags a novel gene predicted by Twinscan as At.chr1.16.98.

To determine the expression pattern of un-annotated genes, we examined reporter gene expression in gene and enhancer trap lines obtained from Cold Spring Harbor Laboratory's Trapper collection [28]. After searching 100 intergenic ORFs against the Trapper database, we identified 19 lines potentially tagging unannotated loci. Expression was reported for two of these lines on the CSHL website. We obtained enhancer or gene trap lines that tagged 3 intergenic loci. With enhancer trap line ET7211, GUS expression was observed in the roots, anthers, pollen, stigma, style, and abscission zones (Figure 5). This enhancer trap insertion is situated proximal to At.chr1.16.98, 407 bp upstream of the start codon. Our experimental sequence corresponding with this locus shows similarity (Expect = 1.7e-17) to a desiccation associated protein from *Lilium longiflorum *and multiple Rop-interactive CRIB (RIC) motif-containing proteins from *Arabidopsis thaliana*, a family of versatile molecular switches involved in many phases of plant development and environmental response [29]. Members of this divergent family of genes have previously been shown to be involved in pollen tube elongation. For line ET1925, which tags Twinscan predicted At.chr1.6.385, we observed GUS expression in developing floral organs (not shown). This enhancer trap is located approximately 600 bp upstream of the At.chr1.6.385 start codon, in the putative promoter region. Additionally, GUS expression for this line has been reported on CSHL's TrapperDB website in trichomes, immature leaves and the epidermis, though we have not observed this pattern. Our experimental sequence for this gene shows high similarity with a putative CLAVATA3/ESR-related (CLE) precursor protein. Due possibly to their small size, genes in the CLE family were overlooked by automated annotation programs [30] but were subsequently annotated upon request. For a third gene, we obtained gene trap line GT5599, which is inserted into an exon of a gene predicted by Twinscan as At.chr1.1.117 (Figure 2). The CSHL website reported low level GUS expression from this line in root tips and root hairs. After examining whole plants ranging in developmental stage from seedling to mature flowering plant, we did not observe any GUS expression with this line, even though our RACE experiments verified that this gene is expressed.

### Promoter-reporter analysis

**Figure 6:**
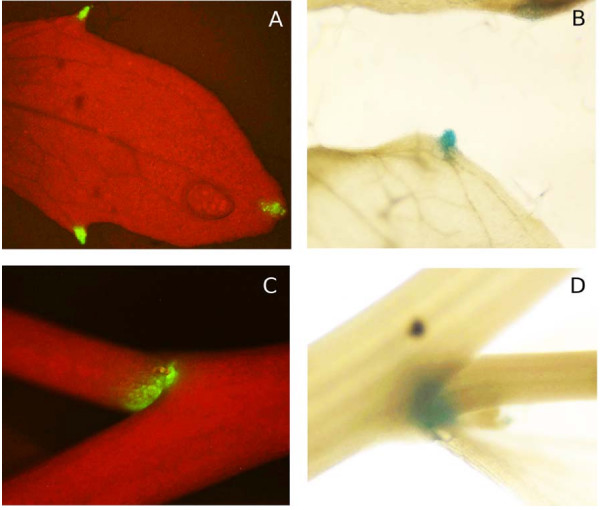
**Promoter-Reporter analysis**. The promoter of intergenic gene At.chr1.15.120 drives expression of the GUS and GFP reporter genes in identical patterns in independent lines generated using two different transformation constructs. Expression of the reporter gene is seen in the hydathode region of leaves (A, B) and at cauline branch junctions (C, D).

We utilized the promoters from six intergenic genes to drive expression of GUS and GFP reporter genes in transgenic Arabidopsis plants. For At.chr1.15.120, (a 345 bp, 2 exon gene for which RACE verified expression) both GUS staining and GFP fluorescence was observed and had consistent patterns in independent transgenic lines. Expression in these lines was localized to the hydathode region of basal leaves, as well as the points to intersection of branching cauline stems (Figure 6). We did not observe GUS staining or GFP fluorescence in transgenic plants containing reporter constructs for the other five intergenic promoters even though RACE results indicate these genes are indeed expressed.

## Conclusion

For the most part, the Arabidopsis genome annotation has relied heavily on the presence of ESTs and FL-cDNA sequences, along with *ab initio *gene predictors such as Genscan [22] and Genemark.hmm [31] to identify the genome's set of protein coding genes. Both Twinscan and EuGene have, in direct ways, used sequence information from related species to expand gene prediction in the Arabidopsis genome. We have used our high-throughput RACE pipeline to assess the reliability of these predictions and have verified the presence of several hundred currently un-annotated genes that were predicted by the Twinscan and/or EuGene programs.

Our decision to use RACE to verify the expression and structure of these un-annotated genes was necessitated by their low level of expression and uncertain gene structures. These genes were not captured by previous large scale EST sequencing efforts and were also not represented in any significant proportion in a normalized Arabidopsis cDNA library sequenced in house (unpublished data). By specifically targeting these most lowly expressed genes with 5' and 3' RACE, we were able to capture transcripts for over a third of the targeted un-annotated genes. However, since our targets excluded many small EuGene predictions of less than 180 bp in length, we can not be certain that the success rates we observed with RACE can be extrapolated to the total set of intergenic predictions. It is unclear whether the remainder of the un-captured targets were not expressed, differed significantly from their predictions, were not present in our cDNA populations at high enough levels to ensure reliable amplification or were not captured due to failure of PCR. In this study, we did not attempt to re-target un-annotated genes with our RACE pipeline, although previous attempts to re-target hypothetical genes by RACE resulted in successful amplification of ~ 40% of the re-targeted genes when using new cDNA populations, suggesting that the relative abundance of signal and the heterogeneity of the cDNA population may likely be a success-limiting factor (unpublished data).

In addition to verifying expression of novel genes by RACE, we have also demonstrated tissue specific activity of intergenic promoters using promoter-reporter fusions, as well as by examining enhancer trap tagged mutants obtained from Cold Spring Harbor Laboratory's Trapper collection. With our promoter-reporter fusions, we observed tissue specific reporter gene expression with one of six promoters tested. With lines obtained from CSHL, we observed reporter gene expression from lines tagging 2 of 3 genes. The lack of expression observed with five of our promoter-reporter lines as well as a gene trap line obtained from CSHL's collection is likely due to either a very low level of expression directed by those promoters, or a very specific pattern, timing, or condition for expression that was not tested by our assays. The pools of cDNA which served as our RACE template originated from several biotic and abiotic treatments that we did not examine in relation to our promoter-reporter constructs.

Overall, we verified the expression and structure of 378 un-annotated genes, 27% of which do not display similarity to any annotated proteins. Furthermore, nearly 50% of the genes described herein are most similar to hypothetical proteins or other proteins of unknown function. Examining the putative functional roles of the remaining un-annotated genes, we can begin to speculate the reasons that many were overlooked by previous annotation efforts. Single copy, lowly expressed genes such as the putative alpha 1,-6, mannosyltransferase corresponding to Twinscan prediction At.chr1.1.117, which does not have similarity with other Arabidopsis genes was likely overlooked due to the lack of support from conserved EST sequences. On the other hand, At.chr1.6.385, which encodes a CLAVATA3/ESR related protein and is a member of a divergent multi-gene family, was likely overlooked due to a combination of lack of EST support [30], and a relatively small coding size. Similarly, we have identified members of a large and divergent gene family encoding Cysteine Rich Peptides. The small size and divergent sequences of this family have contributed to their under-representation in the genome annotation [12]. Additionally, approximately 50% of the top protein hits for the newly verified genes are to hypothetical proteins, or other poorly characterized proteins having unknown function and low expression levels. These examples underscore the limitations of gene prediction programs that still rely to a large extent on training sets derived from relatively abundantly expressed proteins. The incorporation of genomic sequence data from related organisms allows for the easier identification of such genes.

We observed alternative splicing in over 30% of the genes validated with these efforts. This percentage is comparable to that found previously in our work targeting Arabidopsis hypothetical proteins, and higher than the 21.8% of Arabidopsis genes recently reported to be alternatively spliced by Wang and Brendel based upon large scale EST analysis [32]. Our specific RACE-based approach to verifying these genes, using a pool of cDNA from several tissues and treatments resulted in deep sequence coverage and allowed us to capture multiple splicing isoforms. The depth of data that we obtained by sequencing up to 24 clones per gene also allowed us to observe splice isoforms with more regularity than past sequencing efforts. It is uncertain whether the alternatively spliced isoforms identified play a specific biological role, or are simply mis-spliced transcripts.

Both Twinscan and EuGene performed well at identifying un-annotated genes within the Arabidopsis genome. Our success rate for capturing full length sequence information for these genes is in line with our past success in targeting annotated hypothetical proteins. In terms of accuracy, Twinscan and EuGene predicted gene structures with comparable success, though EuGene predicts many smaller, single and multi-exon genes than Twinscan. Based upon the number of intergenic loci predicted by these programs, and our success rates when targeting these loci with 5' and 3' RACE, it is likely that the Arabidopsis genome contains many new genes and other transcribed regions that have yet to be identified.

### TAIR6 annotation

The research and analysis described within this manuscript was carried out with respect to version 5 of the Arabidopsis annotation, as released by TIGR in 2004 (TIGR5). After TIGR's fifth and final release, Arabidopsis annotation responsibility was transferred to TAIR who have recently released an update to the Arabidopsis annotation (TAIR6). This update continued to refine the Arabidopsis annotation using newly submitted EST and cDNA sequences [7]. As a result, 113 out of the 378 genes for which we have full length sequence support are now represented in the TAIR6 annotation. Indeed, with continued sequencing of ESTs and analysis of FL – cDNAs, many of the genes described by this manuscript would eventually be incorporated into future annotation releases, but the use of comparative genomic analysis will greatly speed up the process of identifying and verifying those genes that remain un-annotated.

## Methods

### Gene predictions

Twinscan and EuGene predicted coding sequences (CDS) were obtained from M. Brent and S. Rombauts, respectively. Nomenclature of Twinscan predicted genes is given as At.chrA.B.C. EuGene predicted genes are named as At0XeugYYYZZ. A and X represent the chromosome of the predicted gene. B, C, Y, and Z represent the relative position of the gene prediction on the chromosome.

### Target selection

Twinscan and EuGene predictions were aligned to TIGR5 genome using BLAT. The alignment with the highest identity across the entirelength of the prediction was selected to determine the location of the prediction within the genome. The direction and coordinatesof the alignment were compared to the direction and coordinates of TIGR5 annotated genes in order to determine which predictions were intergenic. A single base pair overlap of a prediction with any annotated gene resulted in that prediction not being considered as intergenic. Initial preference was given to loci predicted by both Twinscan and EuGene. Loci were also targeted that were predicted by only one of the 2 gene prediction programs. When targeting genes predicted only by EuGene, we selected targets having a predicted ORF of larger than 180 base pairs. Twinscan did not predict as many small genes as EuGene, and we thus did not apply a minimum size criterion towards Twinscan predicted genes.

### Primer design

Primer sequences for RACE of intergenic predictions were obtained using an in-house Perl script which designs primers in a batch high-throughput fashion. The script employs MIT primer3 to design and select primers based upon our desired experimental parameters, as described previously [6].

### cDNA synthesis

SMART RACE cDNA populations were prepared as per the manufacturers protocols (Clontech, Mountain View, CA). Two Arabidopsis cDNA populations were generated, one each for 5' and 3' RACE. 5'cDNA populations were prepared using the 5' CDS oligonucleotide, SMART IIA oligonucleotide and PowerScript reverse transcriptase. 3' cDNA populations were prepared using a 3' CDS oligonucleotide.

SMART cDNA populations were generated from 1 μg of PolyA+ RNA pools. These pools contained equal representation of 14 tissue types and treatments including heat shock, cold shock, young plant, Xanthomonas, Pseudomonas, tissue culture suspension, inflorescence, roots, 2,4-Dichlorophenoxyacetic acid treatment, salt stressed, indole acetic acid treatment, UV exposure, and hydrogen peroxide treatment as described previously[6].

### RACE PCR and cloning

RACE was performed on a MJ Research PTC-200 Tetrad thermalcycler. 25 μl reactions contained 2.5 μl 10× PCR buffer, 0.5 μl 100 mM dNTP mix, 0.5 μl PCR Advantage2 Polymerase mix (Clontech), 0.5 μl 10 μM adapter/vector primer, 4 μl 1.25 μM gene specific primer, 0.5 μl template (BD SMART 5' or 3' RACE-ready cDNA).

Cycling conditions were 94 C for 30 sec, followed by 5 cycles of 94 C for 5 sec, 72 C for 4 min, followed by 5 cycles of 94 C for 5 sec, 70 C for 4 min, followed by 25 cycles of 94 C for 5 sec, 68 C for 4 m, a final elongation at 68 C for 5 min. PCR products were visualized on a 1.2% agarose gel stained with ethidium bromide. Successful amplification products were subcloned into the pCR4-TOPO-TA vector (Invitrogen, Carlsbad, CA). Up to 24 clones for each gene were selected and sent for sequencing.

### Data analysis

Sequences of cloned RACE products were trimmed of vector and PolyA tails, and mapped to TIGR5 annotation using the Program to Assemble Spliced Alignments (PASA)[3], along with the Twinscan and EuGene predictions. The PASA user interface and MySQL backend database were used to curate the assembled sequences, examine their locations within the genome and determine whether sufficient experimental evidence existed to verify Twinscan or EuGene predictions.

### Generation of FL ORF clones

If RACE data provided full length coverage of a predicted intergenic gene, or sufficient partial length coverage to support the original or an updated gene model, the full length gene model produced by PASA was extracted, and the gene was re-targeted for full length cloning, as described previously [33]. ORF clones have been deposited with the Arabidopsis Biological Resource Center and are publicly available.

### Structural analysis

An in-house script was used to generate Gene Transfer Format (GTF) files corresponding to the predicted and experimentally validated gene models from BLAT alignments of the CDS to the Arabidopsis genome. The EVAL software package [34] was then used to make comparisons between our experimentally verified intergenic genes and the underlying Twinscan and EuGene predictions. Sensitivity and specificity was determined at a gene level based upon the longest experimentally verified isoform of each gene. A correct prediction is defined as one which a predicted open reading frame is in complete structural agreement with the resulting experimental evidence. For gene level analysis, the longest verified open reading frame from all 378 loci for which we recovered full length sequence support was compared against all Twinscan or EuGene predictions that overlapped those ORFs.

### Functional analysis

To determine the functional nature of the newly verified un-annotated genes, intergenic sequence assemblies were searched using blastx [35] against TIGR's in-house comprehensive non-identical amino acid database, which includes all proteins available from GenBank, PIR, Swiss-Prot, and TIGR's Comprehensive Microbial Resource catalogue, the Omniome. To identify the gene families to which select intergenic genes belong, we made use of multiple sequence alignments generated using the program clustalX [36].

### Enhancer trap tagged arabidopsis

Enhancer and gene trap Arabidopsis lines were obtained from the Cold Spring Harbor Laboratory's Trapper collection (genetrap.cshl.org). Tissues ranging from young seedlings to entire mature plants were assayed with staining buffer containing the chromogenic substrate 5-Bromo-4-chloro-3-indolyl b-D-glucuronic acid (X-gluc).

### Promoter-reporter analysis

To analyze the expression of a selection of intergenic genes, we cloned ~ 2 kb of putative promoter regions upstream of 6 intergenic Twinscan predictions (At.chr1.12.123, At.chr1.14.155, At.chr1.14.398, At.chr1.15.107, At.chr1.15.120, and At.chr1.15.124.) [see additional file 1] and used these promoters to drive *in planta *expression of the GUS and GFP reporter genes using the transformation vectors pYXT1 and pYXT2, as described previously [6]. Plants were grown under 16 hours of light and were assayed for GFP or GUS activity at various developmental stages ranging from seedlings to mature plants.

### Data access

Sequences described have been submitted to GenBank. Submitted sequences are in the accession number range of EF182856 to EF183451.

## Authors' contributions

WAM managed the RACE pipeline, data analysis, and drafted this manuscript. HCW wrote the custom scripts used for primer design and GTF file construction, and carried out other informatic tasks such as sequence mapping. BAU produced FL ORF clones and assisted with data analysis. WW carried out promoter-reporter experiments and analysis. CDT and YX both conceived of the study and its design, and helped to draft the manuscript. All authors have read and approved the final manuscript.

## Supplementary Material

Additional File 1 **Primer sequences used for promoter cloning**. Contains primer sequences used to clone 6 promoters and used for promoter-reporter analysis. Gateway compatible adapter sequences are shown in lowercase and promoter specific sequences are indicated by uppercase letters. Plain text file. Click here for file

## Figures and Tables

**Table 1:** Gene structure statistics for intergenic Twinscan predictions and intergenic EuGene predictions

	***Twinscan***	***EuGene***
# of intergenic predictions	1515	1774
Mean CDS length	342 bp	254 bp
Mean number of exons	1.8	1.5
Percent of single exon genes	854 (56%)	1086 (61%)
Mean CDS length, single-exon predictions	303 bp	260 bp
Mean CDS length, multi-exon	391 bp	245 bp
# of spliced predictions < 100 bp	27	239
# of predictions > 300 bp	608 (40%)	403 (23%)

**Table 2:** Success rate and structural characteristics for RACE-targeted intergenic genes.

**Target Statistics**	*Twinscan*	*EuGene*	*Combined*
Number targeted	726	623	1071*
Mean CDS length	456 bp	354 bp	-
Median CDS length	357 bp	264 bp	-
Average # of exons	2.0	1.4	-
% single exon predictions	423 (58%)	452 (73%)	-

**Experimental Statistics**	*Twinscan*	*EuGene*	*Combined*

Number successful^†^	304 (42%)	257 (41%)	378 (35%)
Mean CDS length	445 bp	452 bp	441 bp
Median CDS length	339 bp	330 bp	321 bp
Average # of exons	1.8	1.7	1.7

* Primer pairs were designed for a total of 1071 total loci. 623 pairs of primers were compatible with a EuGene prediction, and 726 pairs of primers were compatible with a Twinscan prediction. 278 primer pairs were compatible with both a Twinscan and a EuGene prediction.

† For Twinscan and EuGene, success rates are defined as the number of FL sequences obtained that overlap a Twinscan or EuGene prediction, as compared to the number targeted. For the Combined category, the success rate represents the total number of loci verified with respect to the total number targeted.

**Table 3:** Top ten blastx hits among 378 intergenic ORFs.

***Predicted by***	***Best match***	***Description***	***E-value***
At.chr4.1.125/At04eug01370	AAB61038.1	contains similarity to membrane associated salt-inducible protein {Arabidopsis thaliana;}	8.8e-273
At.chr5.6.182/At05eug23610	NP_001031936.1	hydrolase, hydrolyzing O-glycosyl compounds {Arabidopsis thaliana;}	6.5e-247
At.chr5.5.142/At05eug19100	NP_001031908.1	Nucleotidyltransferase {Arabidopsis thaliana;}	1e-240
At.chr1.2.81/At01eug05270	NP_001030977.1	unknown protein {Arabidopsis thaliana;}	1.7e-237
At.chr1.10.7/At01eug36540	AAG51252.1	acetyl-CoA carboxylase, putative; {Arabidopsis thaliana;}	2.2e-214
At.chr1.15.124/At01eug50060	NP_001031203.1	unknown protein {Arabidopsis thaliana;}	1e-202
At.chr5.10.162/At05eug30680	NP_001031973.1	unknown protein {Arabidopsis thaliana;}	3.7e-196
At.chr3.3.273/At03eug12310	AAG51009.1	FKBP-type peptidyl-prolyl cis-trans isomerase, putative {Arabidopsis thaliana;}	3.7e-196
At.chr3.11.252/At03eug36080	NP_001030807.1	unknown protein {Arabidopsis thaliana;}	2.2e-191
At.chr2.1.132/At02eug01230	NP_197902.1	unknown protein {Arabidopsis thaliana;}	1.4e-187
